# Microbiome-derived metabolites in early to mid-pregnancy and risk of gestational diabetes: a metabolome-wide association study

**DOI:** 10.1186/s12916-024-03606-6

**Published:** 2024-10-11

**Authors:** Sita Manasa Susarla, Oliver Fiehn, Ines Thiele, Amanda L. Ngo, Dinesh K. Barupal, Rana F. Chehab, Assiamira Ferrara, Yeyi Zhu

**Affiliations:** 1 School of Public Health, University of California, Berkeley, Berkeley, CA USA; 2 West Coast Metabolomics Center, University of California, Davis, Davis, CA USA; 3 School of Medicine, University of Galway, Galway, Ireland; 4 Ryan Institute, University of Galway, Galway, Ireland; 5 Division of Microbiology, University of Galway, Galway, Ireland; 6 APC Microbiome Ireland, University College Cork, Cork, Ireland; 7 Division of Research, Kaiser Permanente Northern California, Pleasanton, CA 94536 USA; 8 Department of Environmental Medicine and Public Health, Icahn School of Medicine at Mount Sinai, New York, NY USA; 9 Center for Upstream Prevention of Adiposity and Diabetes Mellitus (UPSTREAM), Pleasanton, CA USA; 10 Department of Epidemiology & Biostatistics, University of California, San Francisco, CA USA

**Keywords:** Gestational diabetes, Metabolomics, Microbiome, Pregnancy, Risk prediction

## Abstract

**Background:**

Pre-diagnostic disturbances in the microbiome-derived metabolome have been associated with an increased risk of diabetes in non-pregnant populations. However, the roles of microbiome-derived metabolites, the end-products of microbial metabolism, in gestational diabetes (GDM) remain understudied. We examined the prospective association of microbiome-derived metabolites in early to mid-pregnancy with GDM risk in a diverse population.

**Methods:**

We conducted a prospective discovery and validation study, including a case–control sample of 91 GDM and 180 non-GDM individuals within the multi-racial/ethnic The Pregnancy Environment and Lifestyle Study (PETALS) as the discovery set, a random sample from the PETALS (42 GDM, 372 non-GDM) as validation set 1, and a case–control sample (35 GDM, 70 non-GDM) from the Gestational Weight Gain and Optimal Wellness randomized controlled trial as validation set 2. We measured untargeted fasting serum metabolomics at gestational weeks (GW) 10–13 and 16–19 by gas chromatography/time-of-flight mass spectrometry (TOF–MS), liquid chromatography (LC)/quadrupole TOF–MS, and hydrophilic interaction LC/quadrupole TOF–MS. GDM was diagnosed using the 3-h, 100-g oral glucose tolerance test according to the Carpenter-Coustan criteria around GW 24–28.

**Results:**

Among 1362 annotated compounds, we identified 140 of gut microbiome metabolism origin. Multivariate enrichment analysis illustrated that carbocyclic acids and branched-chain amino acid clusters at GW 10–13 and the unsaturated fatty acids cluster at GW 16–19 were positively associated with GDM risk (FDR < 0.05). At GW 10–13, the prediction model that combined conventional risk factors and LASSO-selected microbiome-derived metabolites significantly outperformed the model with only conventional risk factors including fasting glucose (discovery AUC: 0.884 vs. 0.691; validation 1: 0.945 vs. 0.731; validation 2: 0.987 vs. 0.717; all *P* < 0.01). At GW 16–19, similar results were observed (discovery AUC: 0.802 vs. 0.691, *P* < 0.01; validation 1: 0.826 vs. 0.780; *P* = 0.10).

**Conclusions:**

Dysbiosis in microbiome-derived metabolites is present early in pregnancy among individuals progressing to GDM.

**Supplementary Information:**

The online version contains supplementary material available at 10.1186/s12916-024-03606-6.

## Background

Gestational diabetes mellitus (GDM) is a common metabolic pregnancy complication affecting around 3–14% of pregnancies globally, with increasing prevalence in the recent decades [1, 2]. GDM can lead to a multitude of maternal and child sequalae including, but not limited to, pre-eclampsia and type 2 diabetes in the mother, and macrosomia and obesity later in life in the child [3]. However, the biological underpinnings of GDM remain to be elucidated.

Metabolomics, the ‘omics approach closest to the phenotype, can provide a comprehensive pathophysiologic read-out reflecting endogenous and exogenous chemistries, holding promise for better understanding the pathophysiology of GDM [4]. In particular, microbiome-derived metabolites, the end-products of microbial metabolism, are especially important to study since emerging evidence suggests that microbial dysbiosis in the gut may alter host metabolism, given that the gut microbiome is essential for food digestion, immunity modulation, and metabolic regulation [5, 6]. Identifying microbiome-derived metabolites and their associations with GDM risk may provide biological and mechanistic insights into the microbiome-metabolome-host interactions and uncover phenotypic signatures of GDM. Furthermore, the gut microbiota is malleable and can be altered by diet, physical activity, and other environmental factors [7]. Identifying microbiome-derived metabolite markers for GDM may uncover phenotypic signatures and novel targets for upstream prevention, presenting possibilities for early and precision prevention.

Nascent evidence suggests that pre-diagnostic disturbances in microbiome-derived metabolites have been associated with an increased risk of diabetes in non-pregnant populations [8, 9], but studies among pregnant individuals are lacking. Furthermore, the gut microbiome is malleable and can be altered by diet, physical activity, and other environmental factors [7]. Profiling microbiome-derived metabolites and examining prospective associations with risk of GDM can thus shed light on opportunities for early risk prediction and targeted interventions to mitigate the risk of GDM and adverse sequelae.

To address the important knowledge gaps, we conducted a discovery and validation study to examine the prospective associations of microbiome-derived metabolites in early to mid-pregnancy with risk of GDM among sociodemographically diverse pregnant individuals in a large integrated clinical setting. We further developed and validated machine learning models using multi-metabolite panels for GDM risk prediction.

## Methods

### Study design and population

We conducted a discovery-validation study. The study was approved by the human subjects committee of the Kaiser Foundation Research Institute. Written informed consent was obtained from all participants. The discovery set was from the prospective Pregnancy Environment and Lifestyle Study (PETALS), a longitudinal multi-racial/ethnic cohort study with its study design described in detail elsewhere [10]. Briefly, the participants were drawn from members of Kaiser Permanente Northern California (KPNC), an integrated health care delivery system serving over 4.6 million individuals who are representative of the general population residing in the served geographic area in terms of race and ethnicity, neighborhood-level income, education, and social vulnerability [11]. Pregnant individuals at KPNC are universally (> 97%) screened for GDM with the 50-g, 1-h glucose challenge test around 24–28 weeks of gestation. Following an abnormal screening test result (> 7.8 mmol/L), a diagnostic 100-g, 3-h oral glucose tolerance test (OGTT) is performed after a 12-h fast [12]. GDM is ascertained using the Carpenter-Coustan criteria with at least two plasma glucose values at the OGTT meeting or exceeding thresholds: 1-h 10 mmol/L, 2-h 8.7 mmol/L, and 3-h 7.8 mmol/L [13].

**Fig. 1:**
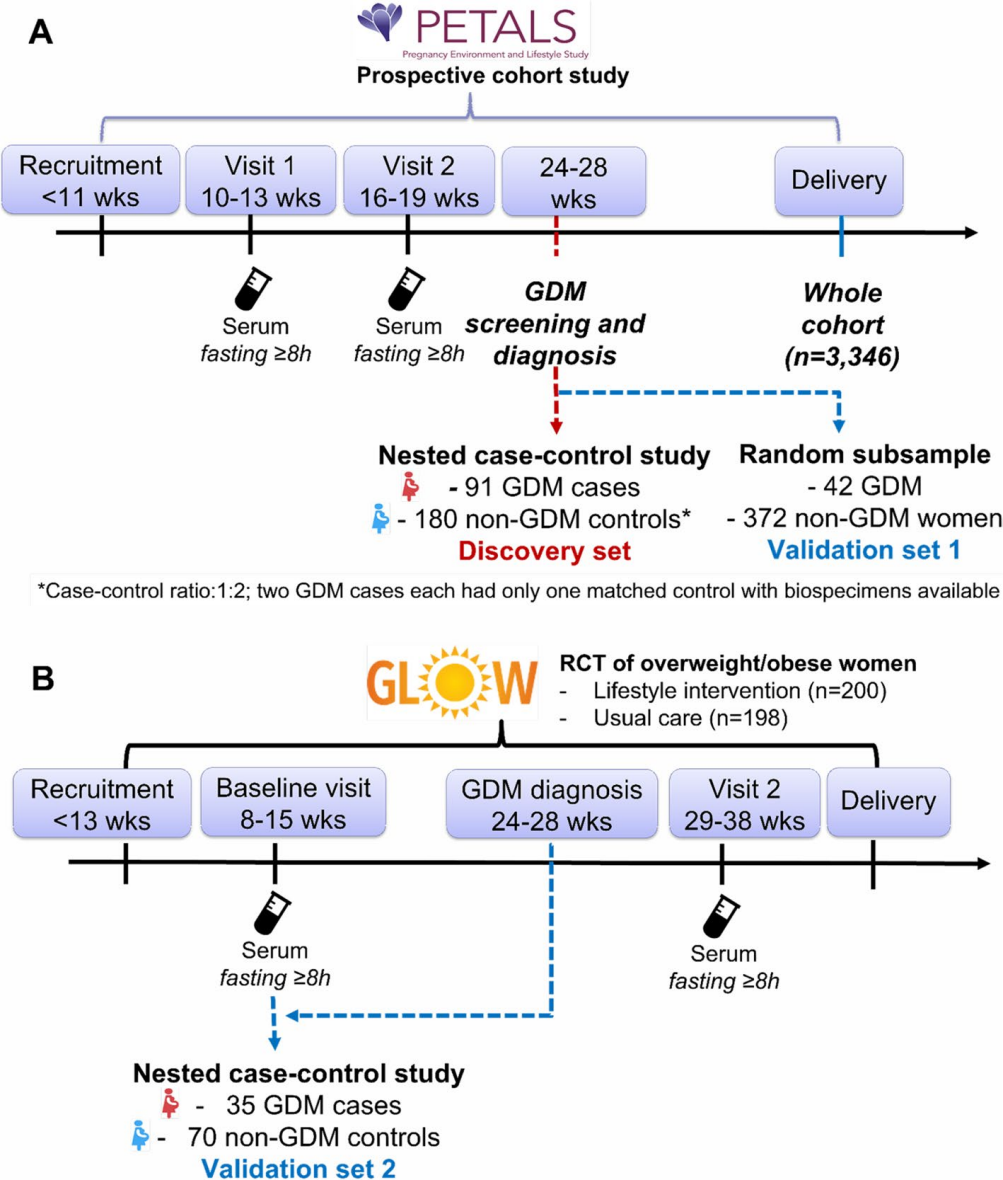
Study flow chart for discovery set and validation sets 1 and 2

In the PETALS cohort, questionnaires on health history and lifestyles were completed at visit 1 (gestational weeks 10–13; baseline), and fasting blood samples were collected after an 8–12-h overnight fast at study clinic visits 1 and 2 (gestational weeks 16–19). To derive the discovery set, we designed a nested case–control study within the PETALS cohort (Fig. 1A). Among participants who delivered between April 2015 and January 2018, we identified 91 GDM cases and 180 non-GDM controls (not 182, with 2 controls missing blood samples) matched 1:2 by age (± 5 years), race/ethnicity, time of enrollment (± 3 months), and gestational weeks at baseline visit (± 3 weeks). Validation set 1 (42 GDM and 372 non-GDM individuals; Fig. 1A) consisted of a 15% random sample of pregnant individuals in the PETALS cohort who delivered between April 2014 and May 2019; had fasting serum samples collected during clinic visits 1 and 2; and were not selected in the discovery set. Validation set 2 was derived using the Gestational Weight Gain and Optimal Wellness (GLOW) randomized controlled trial which aimed to reduce excess gestational weight gain among individuals with pre-pregnancy overweight or obesity through a behavioral lifestyle intervention [14]. The intervention compared to the control group did not affect the incidence of GDM [14]. Pregnant individuals in GLOW with fasting (≥ 8 h) blood samples collected at 8–15 weeks of gestation (baseline visit before the intervention) were included in validation set 2 (35 GDM cases and 70 non-GDM controls matched 1:2 based on the aforementioned factors; Fig. 1B).

### Metabolomics data collection

Fasting serum samples were stored at − 80°C before analysis. Untargeted metabolomics data were obtained at the University of California, Davis West Coast Metabolomics Center. Metabolites from three panels were analyzed via complementary mass spectrometry (MS)-based assays: (1) primary metabolites by gas chromatography/time-of-flight mass spectrometry (TOF–MS) and compound annotation via BinBase database algorithm [15]; (2) complex lipids by liquid chromatography (LC)/quadrupole TOF–MS; and [3] biogenic amines by hydrophilic interaction LC/quadrupole TOF–MS. MS-DIAL version 4.0 software [16] was utilized to process LC–MS data and compounds were annotated based on their accurate mass, retention time, and MS/MS fragment matching using LipidBlast [17] and Massbank.us libraries [18]. For quality control, data were normalized via systematic error removal using random forest [19] to account for batch effects and improve normality. Further, compounds with > 50% missing values in the preprocessing stage for the mass spectrum data and retention index or high technical variance (coefficient of variation > 50%) were removed in the preprocessing stage for the mass spectrum data and retention index. A total of 1362 annotated metabolites met the quality control criteria. We then cross-checked each metabolite against multiple sources and databases—the Virtual Metabolic Human database [20], MetOrigin [21], and Metabolon [8, 22]—to determine whether the metabolite was of gut microbial origin and assembled a list of microbiome-derived metabolites. We identified a total of 140 microbiome-derived metabolites with a coefficient of variation < 20.0% (Additional File 1: Supplemental Table 1). Out of these 140 metabolites, there were missing data on peak intensities for 6 metabolites (missing rate range 0.4–21.4%), which were imputed using the minimum peak intensity of each metabolite divided by 2.

### Statistical analysis

Differences in participant characteristics between cases and controls in the discovery set were assessed by binomial/multinomial logistic regression with generalized estimating equations for binary/multilevel categorical variables, accounting for matched case–control pairs.

For univariate (individual metabolite) analyses, we performed conditional logistic regression to account for case–control matching and to examine the relationships between individual metabolites at gestational weeks 10–13 and 16–19 and risk of GDM, adjusting for covariates. Covariates included age at delivery (continuous), self-identified race/ethnicity (White, Black, Hispanic, Asian/Pacific Islander, and Other/Unknown), race/ethnicity-specific body mass index (Asians: underweight < 18.5 kg/m^2^, normal weight 18.5–22.9, overweight 23.0–27.4, and obese ≥ 27.5; non-Asians: underweight < 18.5 kg/m^2^, normal weight 18.5–24.9, overweight 25.0–29.9, and obese ≥ 30.0), nulliparity (yes/no), pre-existing hypertension (yes/no), and family history of diabetes (yes/no). We also adjusted for gestational week (continuous) and fasting status (yes/no) at the respective clinic visit as precision variables. We used the Benjamini–Hochberg false discovery rate (FDR) method to adjust for multiple testing [23].

For multivariate (multi-metabolites) analyses, we utilized chemical similarity enrichment analysis (ChemRICH) for biochemical cluster mapping and biological interpretation. ChemRICH utilizes structural similarity and chemical ontologies to map all known metabolites and yields study-specific, non-overlapping sets of all identified metabolites [24]. From ChemRICH, we obtained clusters of microbiome-derived metabolites and their associated *P*-values from the Kolmogorov–Smirnov tests [24], which offers a greater statistical power compared to the univariate analysis. Significant pathways were identified based on cluster FDR *P*-values < 0.05. In a sensitivity analysis, we further adjusted for use of prenatal supplements and antibiotics during pregnancy. We also examined changes in metabolites longitudinally from gestational weeks 10–13 to gestational weeks 16–19 in association with risk of GDM.

We further utilized machine learning algorithms to examine the incremental predictive ability of microbiome-derived metabolites beyond conventional risk factors in GDM risk prediction. The following models were constructed: (1) Model 1: aforementioned conventional risk factors for GDM that are well-established in the literature [25] and routinely available in our and potentially many other clinical settings, in addition to fasting serum glucose concentrations given that glucose is routinely used in clinical practice for GDM early detection and risk assessment [26]; (2) Model 2: microbiome-derived metabolite panels at gestational weeks 10–13 and 16–19, respectively; and (3) Model 3: Models 1 and 2 combined. We utilized least absolute shrinkage and selection operation (LASSO) regression which avoids overfitting by regularizing the model and performing feature selection, which leads to parsimonious and more generalizable models, to identify multi-metabolite panels from all microbiome-derived metabolites. Specifically, we employed tenfold cross-validation to identify the lambda parameter that will produce the most optimal LASSO model. We then plotted receiver operating characteristic curves and conducted DeLong’s tests to compare the area under the curve (AUC) statistics. We utilized inverse probability weighting to account for case–control sampling probability to derive results generalizable to the entire PETALS cohort. We further evaluated the model performance of multi-metabolite panels identified in the discovery set in both validation sets 1 and 2.

Data were analyzed using RStudio (version 4.0.3, RStudio PBC, Boston, MA, USA).

### Data resource and availability

Extracted data are available within the publication and its Online Supplemental Material. A de-identified analytic dataset used in this study can be shared with qualified researchers subject to approval by the Kaiser Foundation Research Institute Human Subjects Committee and by the Human Subjects Committee at the institutions requesting the data and a signed data sharing agreement. Please send all requests to the corresponding author.

## Results

### Participant characteristics

In the discovery set (*n* = 271), GDM cases compared to non-GDM cases were more likely to have overweight or obesity before pregnancy, pre-existing hypertension, and family history of diabetes (all *P*-values < 0.05; Table 1). Similar patterns were observed in validation sets 1 and 2 (Additional File 1: Supplemental Table 2). By study design, all participants in validation set 2 had overweight or obesity before pregnancy, representing a higher-risk group for GDM.

**Table 1:** Participant characteristics among all and by gestational diabetes status in the discovery set

	**All (*n* = 271)**	**GDM**	**Non-GDM**^**a**^	***P*-value**^**2**^
		**(*n* = 91)**	**(*n* = 180)**	
**Age at delivery, y, *n* (%)**				0.09
< 25	21 (7.7)	7 (7.7)	14 (7.8)	
25–29	51 (18.8)	13 (14.3)	38 (21.1)	
30–34	122 (45.0)	42 (46.2)	80 (44.4)	
≥ 35	77 (28.4)	29 (31.9)	48 (26.7)	
**Race/ethnicity, *n* (%)**				0.48
White	59 (21.8)	19 (20.9)	40 (22.2)	
Hispanic	89 (32.8)	30 (33.0)	59 (32.8)	
Black	25 (9.2)	5 (5.5)	20 (11.1)	
Asian/Pacific Islander	82 (30.3)	34 (37.4)	48 (26.7)	
Other/unknown	16 (5.9)	3 (3.3)	13 (7.2)	
**Education, *n* (%)**				0.89
High school or less	31 (11.4)	10 (11.0)	21 (11.7)	
Some college	109 (40.2)	37 (40.7)	72 (40.0)	
College graduate or above	131 (48.3)	44 (48.4)	87 (48.3)	
**Parity, *n* (%)**				0.49
0	125 (46.1)	40 (44.0)	85 (47.2)	
1	90 (33.2)	33 (36.3)	57 (31.7)	
2 +	56 (20.7)	18 (19.8)	38 (21.1)	
**Pre-pregnancy BMI, kg/m**^**2**^**, *n* (%)**^**b**^				0.01
Underweight/normal weight	18 (19.8)	64 (35.6)	82 (30.3)	
Overweight	35 (38.5)	54 (30.0)	89 (32.8)	
Obese	38 (41.8)	62 (34.4)	100 (36.9)	
**Pre-existing hypertension, *n* (%)**	16 (5.9)	9 (9.9)	7 (3.9)	0.04
**Family history of diabetes, *n* (%)**	66 (24.4)	34 (37.4)	32 (17.8)	0.001

*BMI*, body mass index; *GDM*, gestational diabetes

^a^Case-control ratio 1:2, with two GDM cases each had only one matched control with biospecimens available

^b^Non-Asians were categorized as underweight (BMI <18.5 kg/m^2^), normal weight (18.5–24.9 kg/m^2^), overweight (25.0–29.9 kg/m^2^), and obese (≥30.0 kg/m^2^). Asians were categorized as underweight (<18.5 kg/m^2^), normal weight (18.5–22.9 kg/m^2^), overweight (23.0–27.4 kg/m^2^), and obese (≥27.5 kg/m^2^). Due to the small number of participants with underweight, this category was combined with normal weight

^2^*P* values for differences were obtained by binomial/multinomial logistic regression with generalized estimating equations for binary/multilevel categorical variables, accounting for matched case-control pairs

### Univariate associations between microbiome-derived metabolites and risk of GDM

The 140 microbiome-derived metabolites were distributed according to metabolic super pathway as follows: benzenoids (4.3%); homogeneous non-metal compounds (0.7%); lipids and lipid-like molecules (20.0%); nucleosides, nucleotides, and analogs (2.1%); organic acids and derivatives (39.3%); organic nitrogen compounds (2.1%); organic oxygen compounds (15.0%); organoheterocyclic compounds (15.0%); and phenylpropanoids and polyketides (1.4%; Additional File 1: Supplemental Fig. 1).

From the univariate conditional logistic regression analysis, 13 microbiome-derived metabolites at gestational weeks 10–13 were positively associated with the risk of GDM, while two were inversely associated with the risk of GDM; and 28 microbiome-derived metabolites at gestational weeks 16–19 were positively associated with GDM, whereas two were inversely associated with GDM (*P*-values < 0.05; Additional File 1: Supplemental Fig. 2; Additional File 1: Supplemental Fig. 3). Univariate associations between individual microbiome-derived metabolites within each super pathway and risk of GDM are visualized in radar plots (Additional File 1: Supplemental Fig. 4). After FDR adjustment, no individual metabolites at gestational weeks 10–13 were significantly associated with GDM risk, whereas seven metabolites at gestational weeks 16–19 (alpha-aminoadipic acid, arachidic acid, glucose, glutamic acid, glycerol, uracil, and uridine) were significantly associated with GDM risk (Additional File 1: Supplemental Fig. 2). In the longitudinal analysis of changes in metabolites from gestational weeks 10–13 to 16–19, 10 metabolites showed positive associations with subsequent risk of GDM whereas one metabolite had a negative association, none of which persisted after FDR adjustment (Additional File 1: Supplemental Fig. 2).

### Multivariate ChemRICH analysis

**Fig. 2:**
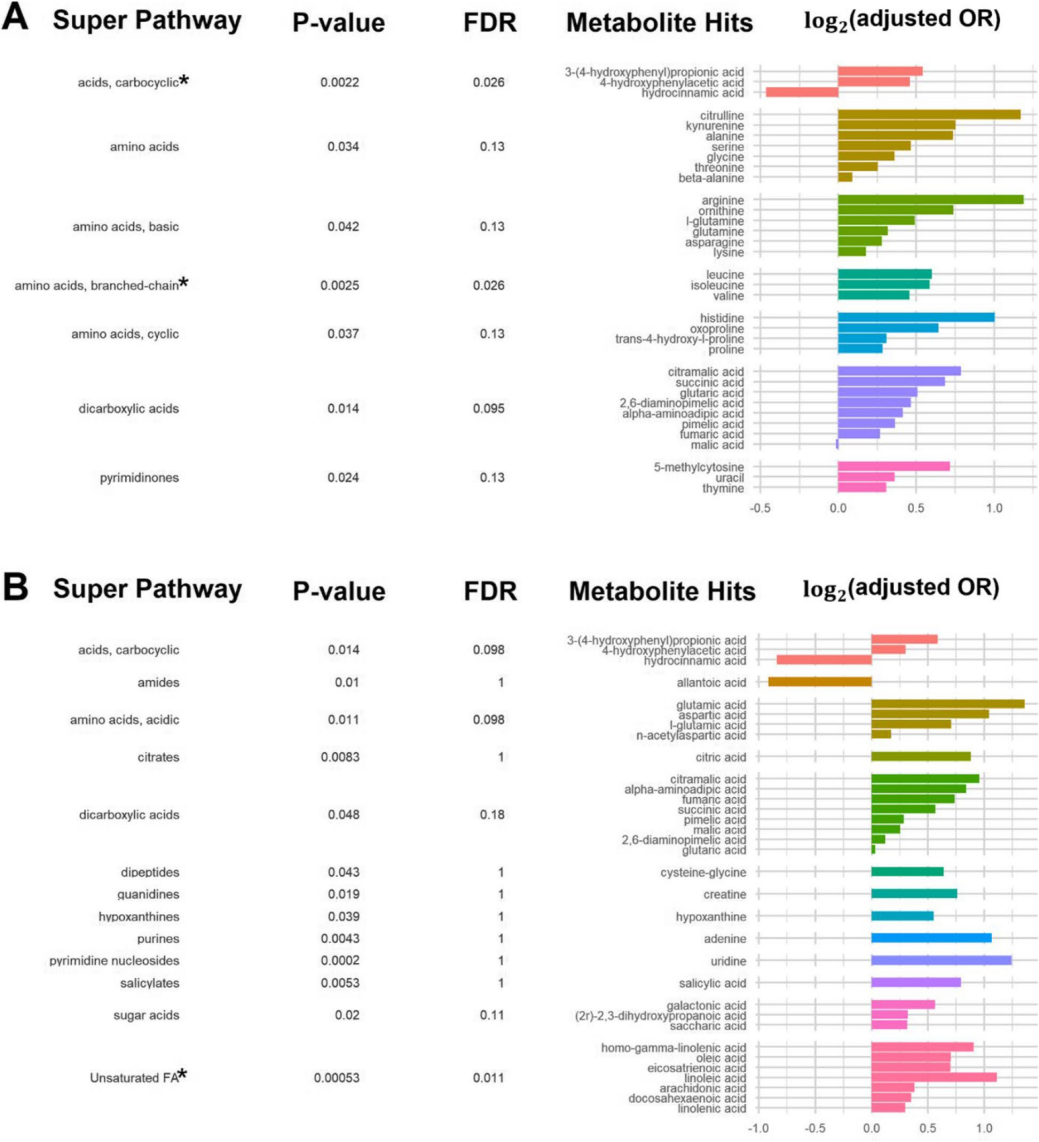
ChemRICH plots depicting the pathways and key metabolites significantly associated with risk of gestational diabetes. **A** 10–13 weeks and (**B**) 16–19 weeks of gestation. ^*^P-value for pathway <0.05 after false discovery rate adjustment

Using the ChemRICH analysis, we identified metabolite clusters or pathways associated with GDM risk (Additional File 1: Supplemental Fig. 5). At gestational weeks 10–13, carbocyclic acids, amino acids, basic amino acids, branched-chain amino acids, cyclic amino acids, dicarboxylic acids, and pyrimidinones clusters were significantly and positively associated with GDM (all *P*-value < 0.05; Fig. 2; Additional File 1: Supplemental Table 3). After FDR adjustment, only the carbocyclic acids and branched-chain amino acids clusters (both FDR = 0.026) remained significantly and positively associated with GDM. At gestational weeks 16–19, carbocyclic acids, acidic amino acids, citrates, dicarboxylic acids, dipeptides, guanidines, hypoxanthines, purines, pyrimidine nucleosides, salicylates, sugar acids, and unsaturated fatty acids were significantly and positively associated with GDM, while amides were significantly and inversely associated with GDM (all *P*-values < 0.05; Fig. 2; Additional File 1: Supplemental Table 3). After FDR adjustment, only the unsaturated fatty acid cluster (FDR = 0.011) remained significantly and positively associated with GDM. In the sensitivity analysis with additional adjustment for prenatal supplements and antibiotics, results remained materially unchanged. When examining changes in metabolites longitudinally from gestational weeks 10–13 to gestational weeks 16–19, sulfur amino acids, guanidines, hexoses, purines, salicylates and unsaturated fatty acids were significantly and positively associated with GDM (all *P*-values < 0.05; Additional File 1: Supplemental Table 3). However, only the unsaturated fatty acids cluster (FDR = 0.011) survived FDR adjustment.

### Multi-metabolite panels for GDM prediction via machine learning

**Fig. 3:**
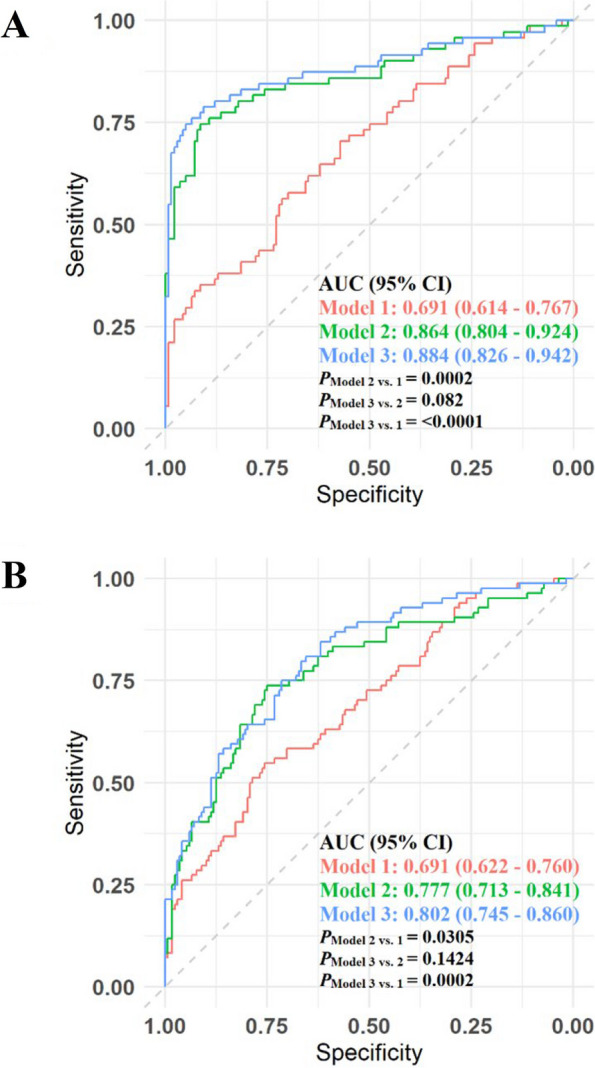
Incremental prediction value of multi-metabolite panels beyond conventional risk factors for gestational diabetes. **A** 10–13 weeks and (**B**) 16–19 weeks of gestation. Receiver operating characteristic (ROC) curves and area under the curve (AUC) statistics were estimated without cross-validation for GDM risk prediction using conventional risk factors (age at delivery, race/ethnicity, pre-pregnancy body mass index, nulliparity, pre-existing hypertension, family history of diabetes, gestational age and fasting status at the respective clinic visit, and fasting serum glucose values; Model 1, red curves); a multi-metabolite panel (Model 2, green curves) selected by least absolute shrinkage and selection operator (LASSO) regression at (**A**) 10–13 weeks and (**B**) 16–19 weeks of gestation; and the selected multi-metabolite panel in addition to conventional risk factors (Model 3, blue curves). *P* values for differences in AUC statistics between models were derived by DeLong’s test

We evaluated the predictability of GDM risk using microbiome-derived metabolites beyond conventional risk factors. At gestational weeks 10–13, LASSO regression identified a 35-metabolite panel (11 amino acids, one aminoxide, one benzenoid, three carbohydrate metabolites, one carbonyl metabolite, one imidazole metabolite, one indolyl carboxylic acid, five lipid metabolites, one nucleoside and nucleotide metabolite, five organic acids, four purine and pyrimidine metabolites, and one tryptamine metabolite; Model 2), which outperformed the predictivity of Model 1 [AUC (95% CI): 0.864 (0.804–0.924) vs. 0.691 (0.614–0.767); *P*_Model 2 vs. 1_ = 0.0002; Fig. 3A; see predictive performance statistics in Additional File 1: Supplemental Table 4 and model optimization in Additional File 1: Supplemental Fig. 6A]. At gestational weeks 16–19, LASSO identified a 11-metabolite panel (one amino acid, one carbohydrate metabolite, one indolyl carboxylic acid, five lipid metabolites, one nucleoside and nucleotide metabolite, one organic acid, and one phenylpropanoid and polyketide metabolite; Model 2) which also outperformed Model 1 [AUC (95% CI): 0.777 (0.713–0.841) vs. 0.691 (0.622–0.760); *P*_Model 2 vs. 1_ = 0.03; Fig. 3B; see predictive performance statistics in Additional File 1: Supplemental Table 4 and model optimization in Additional File 1: Supplemental Fig. 6B].

At gestational weeks 10–13, Model 3 that included a combination of the conventional risk factors and LASSO-selected metabolites significantly outperformed Model 1 [discovery AUC (95% CI): 0.884 (0.826–0.942) vs. 0.691 (0.614–0.767), Fig. 3A; validation 1: 0.945 (0.900–0.990) vs. 0.731 (0.638–0.824); validation 2: 0.987 (0.938–0.999) vs. 0.717 (0.612–0.823), Additional File 1: Supplemental Table 5; all *P* < 0.0001]. At gestational weeks 16–19, similar results were observed [discovery AUC (95% CI): 0.802 (0.745–0.860) vs. 0.691 (0.622–0.760), *P*_Model 3 vs. 1_ = 0.0002, Fig. 3B; validation 1: 0.826 (0.748–0.905) vs. 0.780 (0.688–0.871); *P*_Model 3 vs. 1_ = 0.10, Additional File 1: Supplemental Table 5].

## Discussion

In this prospective discovery and validation study, we examined the associations of microbiome-derived metabolites with risk of GDM. We revealed novel findings on the prospective associations of the carbocyclic acids cluster (key metabolite: 4-hydroxyphenylacetic acid at gestational weeks 10–13 positively associated and hydrocinnamic acid at weeks 16–19 inversely associated) with GDM risk. We also confirmed with the literature other known GDM-associated pathways including branched-chain amino acids and unsaturated fatty acids. Through LASSO machine learning algorithms, we developed and validated predictive multi-metabolite models at gestational weeks 10–13 and 16–19 for GDM risk which exhibited incremental predictability beyond conventional risk factors. Our findings may provide insights into the role of microbiome-metabolome-host interactions in the development of GDM.

Emerging evidence illustrated that microbiome-derived metabolites, not specific microbial species, are associated with blood metabolites of both human and microbial origins as well as gut microbiome diversity [27, 28]. The microbiome-derived metabolome represents the end-products of metabolic activities of the microbiome, which may be functionally more important than the microbial species. Collectively, these findings highlight the importance of focusing on functionally related microbial communities rather than bacterial species per se and the potential of using blood metabolomics to advance the microbiome-host interaction research for biological and mechanistic insights into disease processes. Further, the gut microbiome is malleable and can be altered by exogeneous factors (e.g., diet, physical activity), presenting potential avenues for early disease prevention and intervention [7]. However, previous studies focusing on the role of microbiome-derived metabolites in risk of GDM are lacking.

Our findings revealed novel metabolic pathways and key metabolites implicated in GDM. Through the multivariate enrichment analysis, we observed overall positive association of the carbocyclic acid cluster [hydrocinnamic acid, 3-(4-hydroxyphenyl)propionic acid, 4-hydroxyphenylacetic acid], with 4-hydroxyphenylacetic acid as the key metabolite with positive association at gestational weeks 10–13 and hydrocinnamic acid as the key metabolite with inverse association at weeks 16–19, with risk of GDM. Aromatic amino acids tyrosine and phenylalanine are metabolized into 4-hydroxyphenylacetic acid by colonic bacterial fermentation and then converted into p-cresol, which is then conjugated to glucuronide or sulfate [29, 30]. A study of pregnant individuals at 24–28 weeks of gestation highlighted differentially expressed metabolites in those with GDM including p-cresol and p-cresol sulfate, which were also associated with an increased risk of type 2 diabetes and cardiovascular disease [29, 31]. On the other hand, cinnamic acids and derivatives have been found to have beneficial influence on prevention and management of type 2 diabetes [32]. Although previous comparable data are lacking among pregnant individuals, our finding of the inverse association between hydrocinnamic acid and GDM risk matches prior studies linking hydrocinnamate to a lower risk of type 2 diabetes [33, 34]. Another study found hydrocinnamic acid, a class of food polyphenols, which generally favors the growth of beneficial over pathogenic microorganisms [35], were cross-sectionally and inversely correlated with type 2 diabetes-related microbial features [36]. Stimulating insulin secretion, improving pancreatic β-cell functionality, enhancing glucose uptake, and increasing insulin signaling pathway are some of the putative mechanisms by which cinnamic acids and their derivatives reduce the risk of diabetes [32].

We also detected the dicarboxylic acid cluster (citramalic acid, alpha-aminoadipic acid, fumaric acid, succinic acid, pimelic acid, malic acid, 2,6-diaminopimelic acid, glutaric acid) at both gestational timepoints, with citramalic acid as the key metabolite at gestational weeks 10–13 and alpha-aminoadipic acid as the key metabolite at weeks 16–19, positively associated with GDM risk, although only before FDR adjustment. As a metabolite of yeast or anaerobic bacteria (e.g., Clostridia), citramalic acid is an analog of L-malate, which is an intermediate of carbohydrate metabolism in the citric acid cycle [37]. Despite the lack of comparable previous studies in GDM, animal studies demonstrated elevated levels of citramalic acid and L-malate in the kidneys of diabetic animal models [37]. Alpha-aminoadipic acid, which is a product of lysine degradation and may be modulated by Collinsella [38], has been consistently associated with a higher risk of type 2 diabetes, insulin resistance, and β cell dysfunction [39, 40]. Comparable data in GDM are lacking; however, alpha-aminoadipic acid may be part of a carbonyl stress pathway in diabetes among non-pregnant individuals [40]. Alpha-aminoadipic acid is also hypothesized to modulate insulin secretion and glucose homeostasis by contributing to the initial compensatory upregulation of insulin secretion to maintain glucose homeostasis in early insulin resistance [40].

Beyond recognizing novel microbiome-derived metabolites, our findings extended the literature by confirming several GDM or diabetes-associated metabolic pathways and key metabolites. At gestational weeks 10–13, we found positive association of the branched-chain amino acids (BCAAs) cluster (leucine, isoleucine, valine), with isoleucine as the key metabolite, associated with risk of GDM (FDR < 0.05). Consistently, gut microbiome of insulin-resistant individuals have been shown to have elevated serum levels of BCAAs [31, 41]. Animal models corroborated that *Prevotella corpri* augmented biosynthesis of BCAAs and induced insulin resistance [42]. Specifically, *Prevotella corpri* modulated serum levels of BCAAs may affect the temporary and long-lasting control of insulin secretion and promote hypersecretion, leading to the inhibition of β cell activity [43]. Failure of muscle tissue to properly respond to the anti-catabolic effect of insulin or disruption of BCAA-catabolism have been posed as explanations for the rise in BCAAs levels and their role in insulin resistance [44].

At gestational weeks 16–19, we observed positive association between the unsaturated fatty acid cluster (homo-gamma-linolenic acid, oleic acid, eicosatrienoic acid, linoleic acid, arachidonic acid, docosahexaenoic acid, linolenic acid), with oleic acid as the key metabolite, and risk of GDM (FDR < 0.05). The changes of metabolites in this cluster between gestational weeks 10–13 and 16–19 were also positively correlated with GDM (FDR < 0.05). Blood levels of oleic acid and linoleic acid have been shown to be elevated in other pre-, at-, and post-diagnosis studies of GDM [45–47]. A metabolomics profiling study illustrated elevated levels of oleic acid, linoleic acid, arachidonic acid, stearic acid, palmitic acid, and α-linolenic acid in the placenta of GDM individuals, suggesting the upregulation of the unsaturated fatty acid biosynthesis pathway in GDM [48]. While oleic acid and linoleic acid have previously been reported to have antidiabetic effects and a protective effect against insulin resistance in type 2 diabetes among non-pregnant individuals, there seem to be metabolic abnormalities of unsaturated fatty acids specific to GDM, which may be partially attributable to increased enzyme activity of phospholipase A_2_ during pregnancy as modulated by gut microbiome, resulting in release of these fatty acids from phospholipids [45, 49].

While ChemRICH analysis focused on biochemical pathway mapping based on chemical similarity enrichment to facilitate biological interpretation, predictive models via machine learning algorithms may help identify markers with predictive value for GDM risk. Through the LASSO algorithm, we observed panels of multi-microbiome-derived metabolites had predictive value beyond conventional risk factors. We discovered 35 microbiome-derived metabolites at gestational weeks 10–13 and 11 metabolites at weeks 16–19 illustrating predictive value for GDM risk. Of these, we identified four novel metabolic markers including trans-4-hydroxy-l-proline, 4-imidazoleacrylic acid, alpha-keto-gamma-(methylthio)butyric acid, and 2,6-diaminopimelic acid with incremental predictive value of GDM risk beyond conventional risk factors. Trans-4-hydroxy-l-proline is a major component of collagen and precursor for the synthesis of glycine, pyruvate, and glucose [50]. Due to its high concentration of glycine, collagen has been shown to stimulate insulin secretion and stabilize blood sugar levels in individuals with type 2 diabetes [51]. Moreover, glycine levels have been inversely correlated with insulin resistance and obesity and have been consistently low in individuals with type 2 diabetes [52]. 4-imidazoleacrylic acid, also known as trans-urocanic acid, is involved in histidine metabolism. A randomized controlled trial of women with obesity and metabolic syndrome found histidine suppressed inflammation and oxidative stress and improved insulin resistance [53]. Consistently, a case–control study found individuals with type 2 diabetes and microalbuminuria had lower levels of histidine [54]. Alpha-keto-gamma-(methylthio)butyric acid, synthesized from L-methionine and butyric acid, is the keto form of L-methionine. A case–control study found GDM versus non-GDM individuals had higher plasma concentrations of methionine [55]. Experimental studies in humans and rodents also illustrated methionine restriction as a method of improving insulin sensitivity and glucose homeostasis [56, 57]. Finally, 2,6-diaminopimelic acid, as an Escherichia coli metabolite, is a carboxylic acid. Diaminopimelic acid is a key component of the bacterial cell wall and typically found in human urine and feces due to enzymatic breakdown of gut microbiome. Under conditions of chronic low-grade inflammation in obesity and type 2 diabetes induced by microbial dysbiosis, diaminopimelic acid activates nucleotide-binding oligomerization domain-containing protein 1 which stimulates insulin resistance and insulin trafficking in β cells [58].

Our study has several notable strengths. First, our research question is novel by focusing on microbiome-derived metabolites, the end-products of microbial metabolism. There is emerging evidence that the gut microbiome plays a role in various metabolic diseases by altering the concentration of metabolites via microbiome-related metabolism and microbiome-host co-metabolism [27]. We used the untargeted metabolomics approach to better understand the microbiome-metabolome-host metabolism. Second, the use of fasting serum minimized measurement variability due to fasting status. Third, the diverse individuals in our sample with one discovery and two validation sets ensure robustness of our findings. Finally, to minimize misclassification, we used standardized clinical diagnosis of GDM using the Carpenter-Coustan criteria for both the discovery and validation sets.

Some limitations are also to be noted. Although serum samples were collected before GDM diagnosis, given the observational nature of the study, causality cannot be established. Whether metabolites significantly associated with GDM risk were causal molecules or signatures of a pre-diagnostic pathophysiologic state remains to be elucidated via functional studies. We did not directly measure the microbial species involved in GDM; however, we profiled fasting serum untargeted metabolomics, reflecting the end-products of gut microbiome metabolism, which could be metabolically more important than individual microbial species. Despite rigorous validation using two separate validation sets, our multi-metabolite panels for GDM risk prediction warrant further validation in other populations and clinical settings. Further, future research focusing on simplifying the model without compromising its predictive accuracy is needed to improve the clinical translational significance.

## Conclusions

In this discovery-validation study, we focused on microbiome-derived metabolites to provide specific insights into the role of microbiome-metabolome-host interactions in GDM. We found dysbiosis of the microbiome-derived metabolome measured in fasting serum in early to mid-pregnancy among individuals who later developed GDM. For the first time, we illustrated the significant associations of the carbocyclic acids (key metabolites: 4-hydroxyphenylacetic acid and hydrocinnamic acid) in early to mid-pregnancy with risk of GDM. The machine learning algorithm identified multi-metabolite panels that indicate a combination of these metabolites with the conventional risk factors may improve risk prediction of GDM beyond conventional risk factors. Investigating these microbiome-derived metabolites may help identify future opportunities for intervention and prevention of GDM. Further research is warranted to confirm our findings and better understand the microbiome-metabolome-host interactions and the roles these metabolites play in the development of GDM. If findings are confirmed, they may inform novel preventative strategies that leverage the malleability of the gut microbiota to mitigate the risk of GDM.

## Supplementary Information

Additional file 1: Supplemental Table 1. Microbiome-derived metabolites included in statistical analysis by super pathway. Supplemental Table 2. Participant characteristics in the validation sets 1 (a random sample in the PETALS cohort) and 2 (a nested case-control study within the GLOW trial). Supplemental Figure 1. Tree map showing the distributions of the microbiome-derived metabolites according to their super pathway and related sub-pathways. Supplemental Figure 2. Univariate forest plots depicting individual metabolites at A) 10-13 and B) 16-19 weeks of gestation and C) changes in metabolites from 10-13 to 16-19 weeks of gestation significantly associated with the risk of gestational diabetes. Supplemental Figure 3. Univariate volcano plots depicting microbiome-derived metabolites positively associated and inversely associated with risk of gestational diabetes at A) 10-13 and B) 16-19 weeks of gestation. Supplemental Figure 4. Radar plots depicting univariate associations between individual microbiome-derived metabolites within each super pathway at A) 10-13 and B) 16-19 weeks of gestation and risk of gestational diabetes. Supplemental Figure 5. Multivariate ChemRICH enrichment plots depicting all pathways identified at A) 10-13 and B) 16-19 weeks of gestation. Supplemental Table 3. The putative pathways linking microbiome-derived metabolites at 10-13 and 16-19 weeks of gestation (GW) to risk of gestational diabetes using the multivariate ChemRICH analysis. Supplemental Table 4. Predictive performance of multi-metabolite panels at 10-13 weeks and 16-19 weeks of gestation beyond conventional risk factors for gestational diabetes using LASSO regression models. Supplemental Figure 6. Model optimization of LASSO regression models for the selection of multi-metabolite panels at A) 10-13 and B) 16-19 weeks of gestation in the discovery set. Supplemental Table 5. External validation of predictive multi-metabolite panels at 10-13 weeks and 16-19 weeks of gestation beyond conventional risk factors for gestational diabetes using LASSO regression models.

## Data Availability

Extracted data are available within the publication and its appendix. A de-identified analytic dataset used in this study can be shared with qualified researchers subject to approval by the Kaiser Foundation Research Institute Human Subjects Committee and by the Human Subjects Committee at the institutions requesting the data and a signed data sharing agreement. Please send all requests to the corresponding author of this article.

## Abbreviations

AUC: Area under the curve
BCAAs: Branched-chain amino acids
ChemRICH: Chemical similarity enrichment analysis
FDR: False discovery rate
GDM: Gestational diabetes mellitus
GLOW: Gestational Weight Gain and Optimal Wellness
KPNC: Kaiser Permanente Northern California
LASSO: Least absolute shrinkage and selection operation
LC: Liquid chromatography
MS: Mass spectrometry
OGTT: Oral glucose tolerance test
PETALS: Pregnancy Environment and Lifestyle Study
TOF-MS: Time-of-flight mass spectrometry

## Data availability

No datasets were generated or analysed during the current study.
