# Supplementary material for: Microbiome-derived metabolites in early to mid-pregnancy and risk of gestational diabetes: a metabolome-wide association study
Source: BMC Med. 2024 Oct 11;22:449. doi: 10.1186/s12916-024-03606-6 (PMC11470649; doi:10.1186/s12916-024-03606-6)
Supplement: Supplementary file 1 — Additional file 1: Supplemental Table 1. Microbiome-derived metabolites included in statistical analysis by super pathway. Supplemental Table 2. Participant characteristics in the validation sets 1 (a random sample in the PETALS cohort) and 2 (a nested case-control study within the GLOW trial). Supplemental Figure 1. Tree map showing the distributions of the microbiome-derived metabolites according to their super pathway and related sub-pathways. Supplemental Figure 2. Univariate forest plots depicting individual metabolites at A) 10-13 and B) 16-19 weeks of gestation and C) changes in metabolites from 10-13 to 16-19 weeks of gestation significantly associated with the risk of gestational diabetes. Supplemental Figure 3. Univariate volcano plots depicting microbiome-derived metabolites positively associated and inversely associated with risk of gestational diabetes at A) 10-13 and B) 16-19 weeks of gestation. Supplemental Figure 4. Radar plots depicting univariate associations between individual microbiome-derived metabolites within each super pathway at A) 10-13 and B) 16-19 weeks of gestation and risk of gestational diabetes. Supplemental Figure 5. Multivariate ChemRICH enrichment plots depicting all pathways identified at A) 10-13 and B) 16-19 weeks of gestation. Supplemental Table 3. The putative pathways linking microbiome-derived metabolites at 10-13 and 16-19 weeks of gestation (GW) to risk of gestational diabetes using the multivariate ChemRICH analysis. Supplemental Table 4. Predictive performance of multi-metabolite panels at 10-13 weeks and 16-19 weeks of gestation beyond conventional risk factors for gestational diabetes using LASSO regression models. Supplemental Figure 6. Model optimization of LASSO regression models for the selection of multi-metabolite panels at A) 10-13 and B) 16-19 weeks of gestation in the discovery set. Supplemental Table 5. External validation of predictive multi-metabolite panels at 10-13 weeks and 16-19 weeks of gestation beyo [file 12916_2024_3606_MOESM1_ESM.docx]

| **Supplemental Table 1: Microbiome-derived metabolites included in statistical analysis by super pathway** | | |
| --- | --- | --- |
| **Benzenoids** | Asparagine | Cellobiose |
| 2-Methylbenzyl Alcohol | Aspartic Acid | Fructose |
| 4-Hydroxyphenylacetic Acid | Beta-Alanine | Fucose |
| Hippuric Acid | Betaine | Glucose |
| Hydroquinone | Citric Acid | Glyceric Acid |
| P-Cresol | Citrulline | Glycerol |
| Salicylic Acid | Creatine | Kynurenine |
| **Homogeneous Non-Metal Compounds** | Creatinine | Lactose |
| Pyrophosphate | Cysteine | Lyxose |
| **Lipids and Lipid-like Molecules** | Cysteine-Glycine | Melibiose |
| 3-Hydroxybutyrylcarnitine | Cystine (DL) | Myo-Inositol |
| Alpha-Keto-Gamma-(Methylthio)Butyric Acid | Fumaric Acid | N-Acetylmannosamine |
| Arachidic Acid | Galactonic Acid | Pantothenic Acid |
| Arachidonic Acid | Glutamic Acid | Ribose |
| Cholesterol | Glutamine | Saccharic Acid |
| Citramalic Acid | Glutaric Acid | Sucrose |
| Docosahexaenoic Acid | Glycine | Xylitol |
| Eicosatrienoic Acid | Glycolic Acid | Xylose |
| Glycerophosphocholine | Histidine | **Organoheterocyclic Compounds** |
| Glycocholic Acid | Homocysteine | 4-Imidazoleacrylic Acid |
| Homo-Gamma-Linolenic Acid | Indoxyl Sulfate | 5,6-Dihydrouracil |
| Linoleic Acid (Positive) | Isoleucine | 5-Methylcytosine |
| Linoleic Acid (Negative) | Lactic Acid | Adenine |
| Linolenic Acid | Leucine | Caffeine |
| LPC 16:0 | L-Glutamic Acid | DL-Indole-3-Lactic Acid |
| LPC 18:0 | L-Glutamine | Hypoxanthine |
| LPC 18:1 | Lysine | Indole-3-Acetate |
| LPC 18:2 | Malic Acid | Indole-3-Propionic Acid |
| LPC 18:3 | Methionine | L-Tryptophan |
| LPE 16:0 | Methioninesulfoxide | Melatonin |
| LPE 18:0 | N8-Acetylspermidine | Nicotinamide |
| LPE 18:2 | N-Acetylaspartic Acid | Nicotinic Acid |
| LPE 20:4 | N-Acetylputrescine | Serotonin |
| L-Propionylcarnitine | Ornithine | Thymine |
| Myristic Acid | Oxoproline | Tryptamine |
| Octanoylcarnitine | Phenylacetylglutamine | Tryptophan |
| Oleic Acid | Phenylalanine | Uracil |
| Pimelic Acid | Proline | Uric Acid |
| **Nucleosides, Nucleotides, and Analogues** | Serine | Urobilin |
| 5'-S-Methyl-5'-Thioadenosine | Succinic Acid | Xanthine |
| Inosine | Threonine | **Phenylpropanoids and Polyketides** |
| Uridine | Trans-4-Hydroxy-L-Proline | 3-(4-Hydroxyphenyl)Propionic Acid |
| **Organic Acids and Derivatives** | Tyrosine | Hydrocinnamic Acid |
| 2,6-Diaminopimelic Acid | Urea |  |
| 2-Aminobutyric Acid | Valine |  |
| 2-Hydroxybutanoic Acid | **Organic Nitrogen Compounds** |  |
| 3-Hydroxybutyric Acid | Choline |  |
| Aconitic Acid | Betaine Aldehyde |  |
| Alanine | Trimethylamine N-Oxide (TMAO) |  |
| Allantoic Acid | **Organic Oxygen Compounds** |  |
| Alpha-Aminoadipic Acid | Alpha, Beta-Trehalose |  |
| Alpha-Ketoglutarate | Arabinose |  |
| Arginine | Arabitol |  |

| **Supplemental Table 2. Participant characteristics in the validation sets 1 (a random sample in the PETALS cohort) and 2 (a nested case-control study within the GLOW trial)** | | | | | | | | | |
| --- | --- | --- | --- | --- | --- | --- | --- | --- | --- |
|  | **Validation set 1** | | | |  | **Validation set 2** | | | |
|  | **All** | **GDM** | **Non-GDM** | **P-value^1^** |  | **All** | **GDM** | **Non-GDM** | **P-value^1^** |
|  | **(n=414)** | **(n=42)** | **(n=372)** |  |  | **(n=105)** | **(n=35)** | **(n=70)** |  |
| **Age at delivery, y, n (%)** |  |  |  | 0.59 |  |  |  |  | 0.28 |
| <25 | 52 (12.6) | 3 (7.1) | 49 (13.2) |  |  | 2 (1.9) | 2 (5.7) | 0 (0.0) |  |
| 25-29 | 108 (26.1) | 10 (23.8) | 98 (26.3) |  |  | 5 (4.8) | 1 (2.9) | 4 (5.7) |  |
| 30-34 | 160 (38.6) | 17 (40.5) | 143 (38.4) |  |  | 63 (60.0) | 21 (60.0) | 42 (60.0) |  |
| ≥35 | 94 (22.7) | 12 (28.6) | 82 (22.0) |  |  | 35 (33.3) | 11 (31.4) | 24 (34.3) |  |
| **Race/ethnicity, n (%)** |  |  |  | 0.05 |  |  |  |  |  |
| White | 105 (25.4) | 9 (21.4) | 96 (25.8) |  |  | 15 (14.3) | 5 (14.3) | 10 (14.3) | 1.00 |
| Hispanic | 105 (25.4) | 8 (19.0) | 97 (26.1) |  |  | 27 (25.7) | 9 (25.7) | 18 (25.7) |  |
| Black | 99 (23.9) | 7 (16.7) | 92 (24.7) |  |  | 3 (2.9) | 1 (2.9) | 2 (2.9) |  |
| Asian/Pacific Islander | 105 (25.4) | 18 (42.9) | 87 (23.4) |  |  | 42 (40.0) | 14 (40.0) | 28 (40.0) |  |
| Other/unknown |  |  |  |  |  | 18 (17.1) | 6 (17.1) | 12 (17.1) |  |
| **Education, n (%)** |  |  |  | 0.80 |  |  |  |  | 0.08 |
| High school or less | 53 (12.8) | 4 (9.5) | 49 (13.2) |  |  | 7 (6.7) | 5 (14.3) | 2 (2.9) |  |
| Some college | 151 (36.6) | 16 (38.1) | 135 (36.4) |  |  | 17 (16.2) | 6 (17.1) | 11 (15.7) |  |
| College graduate or above | 209 (50.6) | 22 (52.4) | 187 (50.4) |  |  | 81 (77.1) | 24 (68.6) | 57 (81.4) |  |
| **Nulliparity, n (%)** | 185 (44.7) | 18 (42.9) | 167 (44.9) | 0.80 |  | 51 (48.6) | 19 (54.3) | 32 (45.7) | 0.41 |
| **Pre-pregnancy BMI, kg/m^2^, n (%)**^2^ |  |  |  | 0.01 |  |  |  |  | 0.04 |
| Underweight/normal weight | 160 (38.6) | 9 (21.4) | 151 (40.6) |  |  | - | - | - |  |
| Overweight | 115 (27.8) | 10 (23.8) | 105 (28.2) |  |  | 71 (67.6) | 19 (54.3) | 52 (74.3) |  |
| Obese | 139 (33.6) | 23 (54.8) | 116 (31.2) |  |  | 34 (32.4) | 16 (45.7) | 18 (25.7) |  |
| **Chronic hypertension, n (%)** | 26 (6.3) | 2 (4.8) | 24 (6.5) | 0.67 |  | 1 (1.4) | 0 (0.0) | 1 (2.9) | 1.00 |
| **Family history of diabetes, n (%)** | 95 (22.9) | 12 (28.6) | 83 (22.3) | 0.36 |  | 17 (16.2) | 11 (31.4) | 6 (8.6) | 0.003 |
| **GLOW trial arms** |  |  |  |  |  |  |  |  |  |
| Intervention | NA | NA | NA |  |  | 55 (52.4) | 18 (51.4) | 37 (52.9) | 0.89 |
| Usual care | NA | NA | NA |  |  | 50 (47.6) | 17 (48.6) | 33 (47.1) |  |
| BMI, body mass index; GDM, gestational diabetes; GLOW, Gestational Weight Gain and Optimal Wellness; NA, not applicable | | | | | | | | | |
| **^1^***P* values for differences between women with and without GDM were obtained by student's t-test for continuous variables and Chi-square or Fisher's exact test for categorical variables | | | | | | | | | |
| ^2^Non-Asians were categorized as underweight (BMI <18.5 kg/m^2^), normal weight (18.5–24.9 kg/m^2^), overweight (25.0–29.9 kg/m^2^), and obese (≥30.0 kg/m^2^). Asians were categorized as underweight (<18.5 kg/m^2^), normal weight (18.5–22.9 kg/m^2^), overweight (23.0–27.4 kg/m^2^), and obese (≥27.5 kg/m^2^) | | | | | | | | | |

**Supplemental Figure 1: Tree map showing the distributions of the microbiome-derived metabolites according to their super pathway and related sub-pathways^*^**


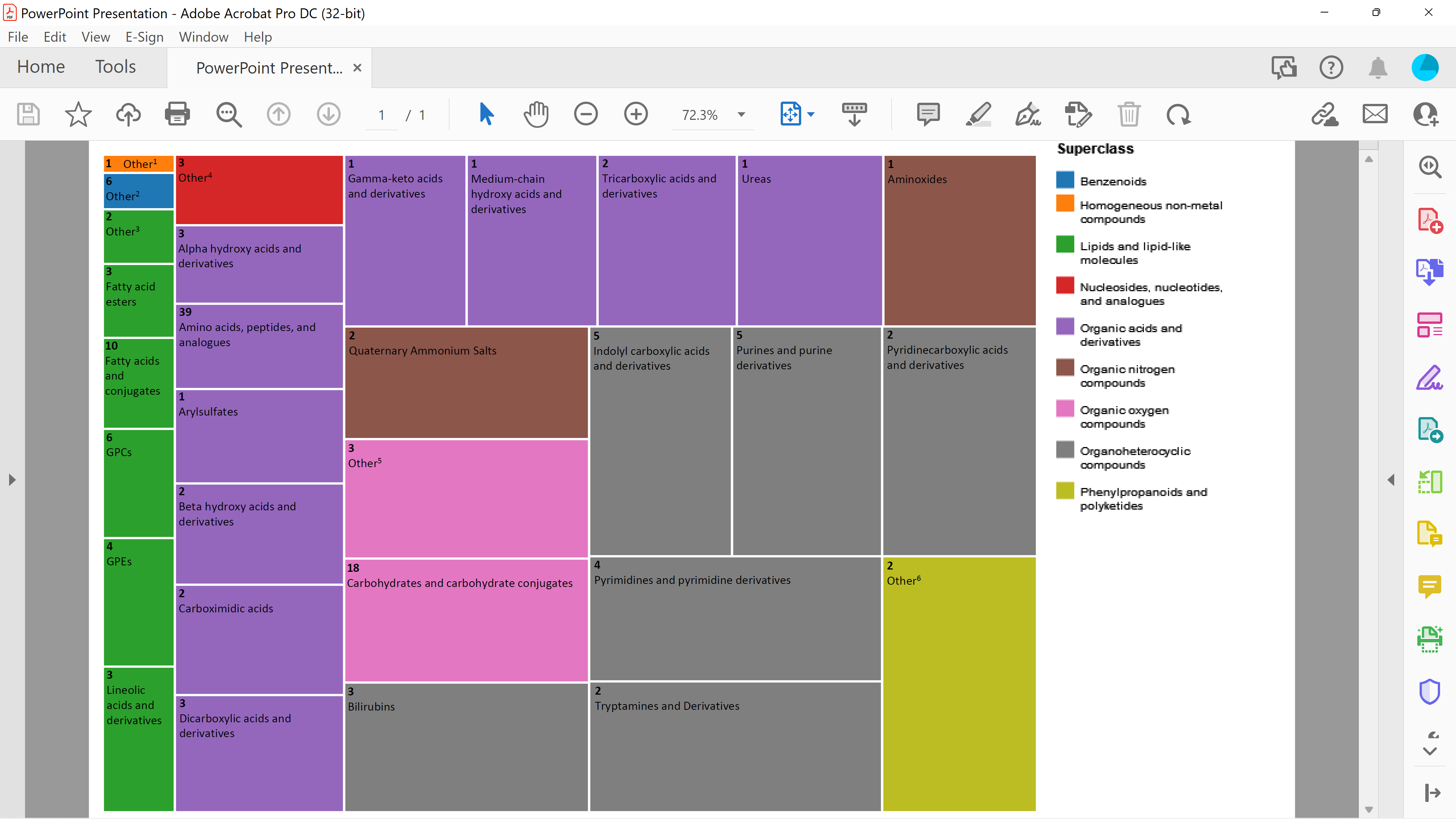


**^*^**Super pathways indicated by color; sub-pathways indicated by the square and the number in each square corresponds to the number of microbiome-derived metabolites within each sub-pathway

**^1^**Non-metal pyrophosphates (1)

**^2^**1-hydroxy-2-unsubstituted benzenoids (1), Benzenediols (1), Benzoic acids and derivatives (2), Benzyl alcohols (2)

**^3^**Cholestane steroids (1), Bile acids, alcohols and derivatives (1)

**^4^**5'-deoxy-5'-thionucleosides (1), NA, Nucleosides, nucleotides, and analogues (2)

**^5^**Alcohols and polyols (2), Carbonyl compounds (1)

**^6^**NA, Phenylpropanoids and polyketides (2)

**Supplemental Figure 2: Univariate forest plots depicting individual metabolites at A) 10-13 and B) 16-19 weeks of gestation and C) changes in metabolites from 10-13 to 16-19 weeks of gestation significantly associated with the risk of gestational diabetes^1^**

**
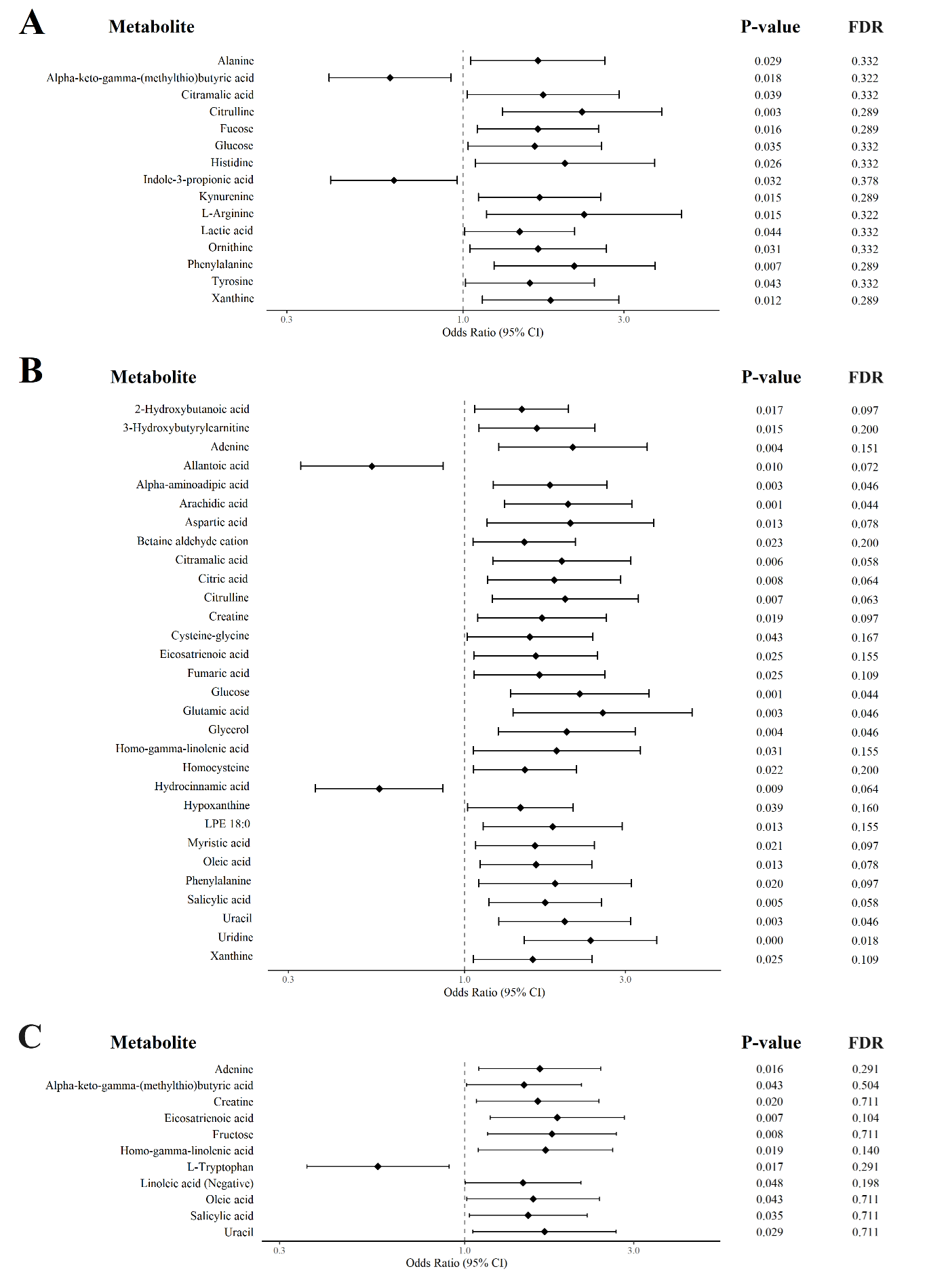
**

**^1^**Adjusted for age at delivery, race/ethnicity, pre-pregnancy body mass index, nulliparity, pre-existing hypertension, family history of diabetes, and gestational age and fasting status at the respective clinic visit.

**Supplemental Figure 3: Univariate volcano plots depicting microbiome-derived metabolites positively associated and inversely associated with risk of gestational diabetes at A) 10-13 and B) 16-19 weeks of gestation^1^**

**
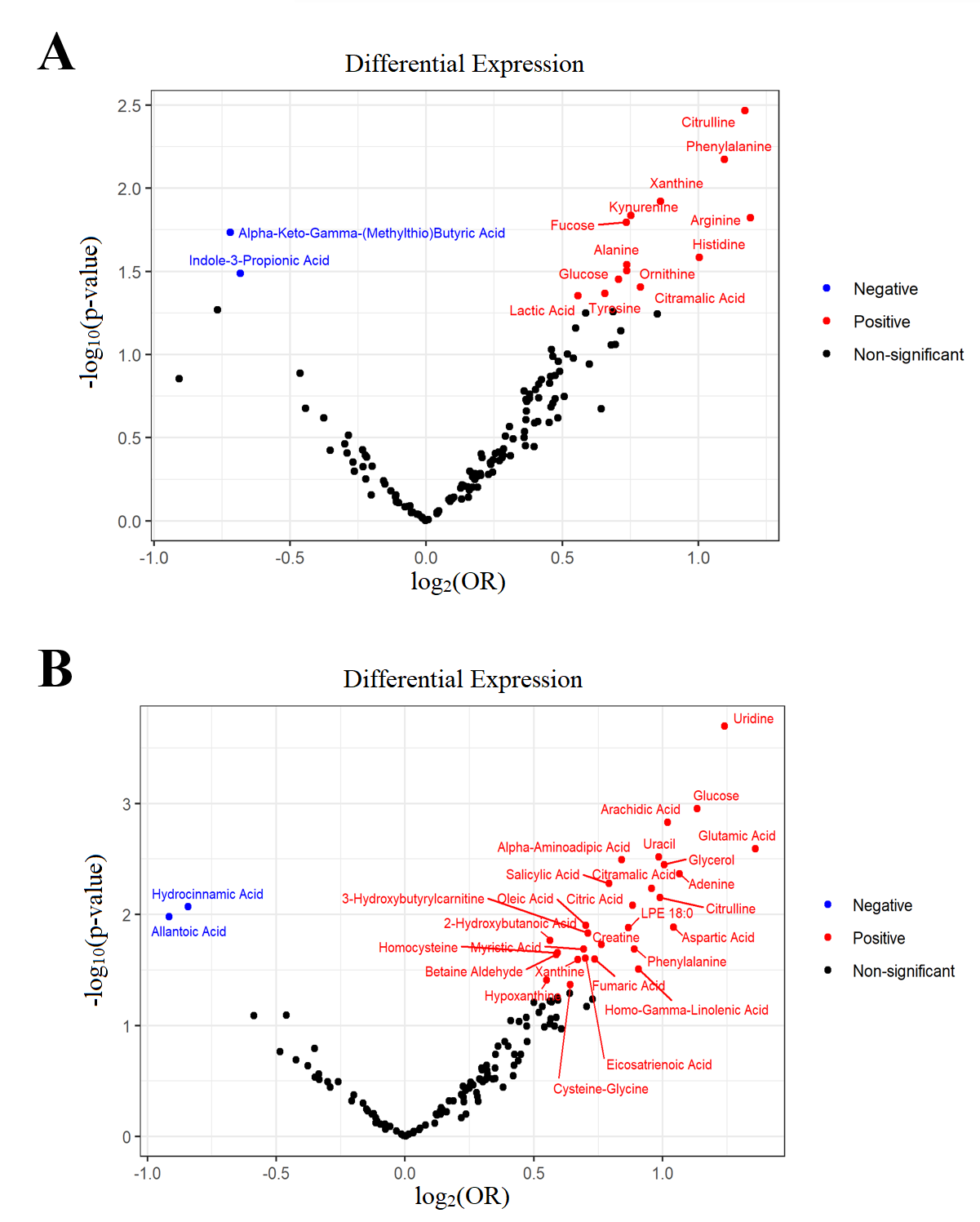
**

**^1^**Adjusted for age at delivery, race/ethnicity, pre-pregnancy body mass index, nulliparity, pre-existing hypertension, family history of diabetes, and gestational age and fasting status at the respective clinic visit

**Supplemental Figure 4: Radar plots depicting univariate associations between individual microbiome-derived metabolites within each super pathway at A) 10-13 and B) 16-19 weeks of gestation and risk of gestational diabetes^1^**


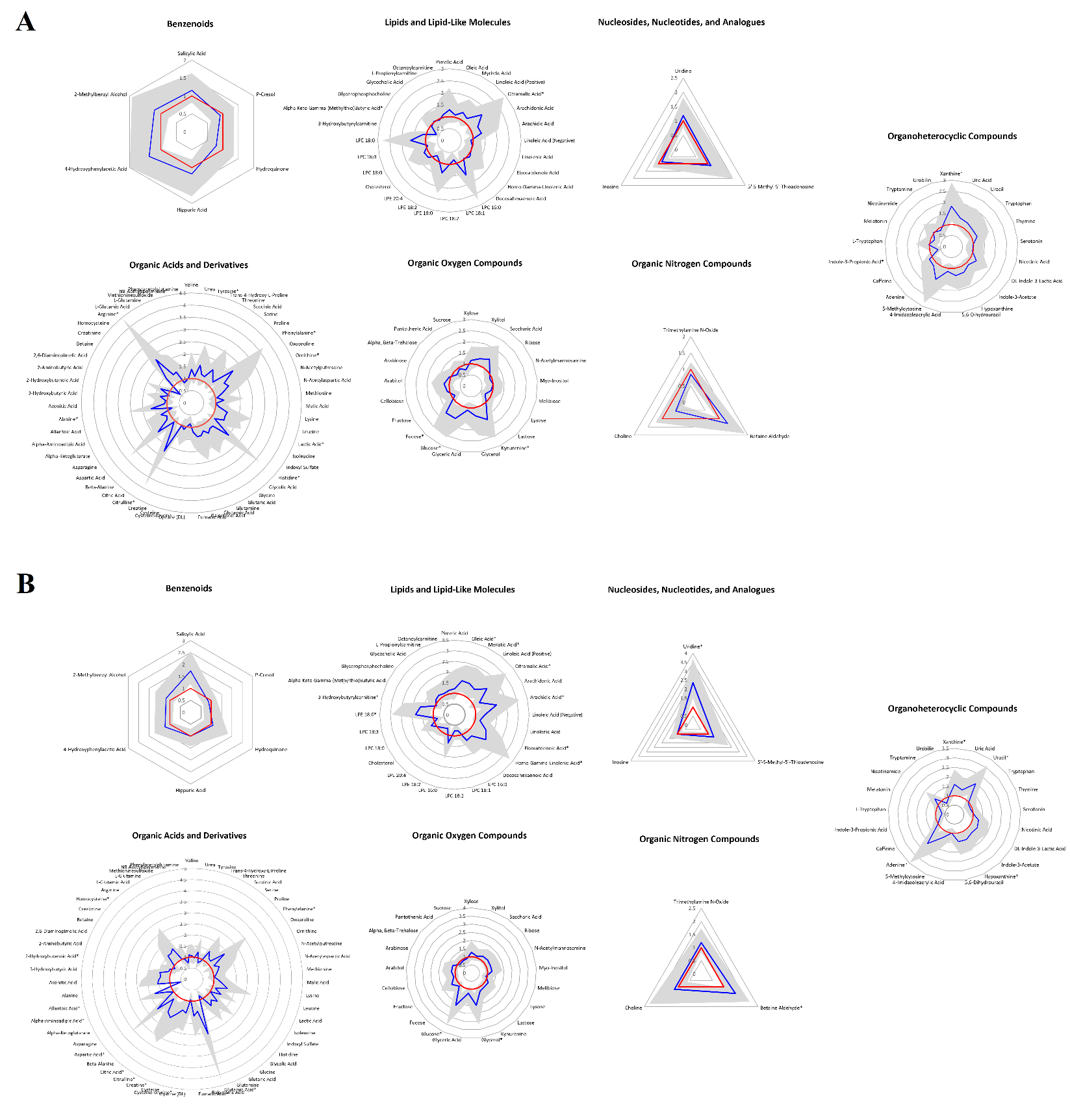


**^*^**P-value for pathway <0.05 after false discovery rate adjustment

**^1^**Red circle represents the null OR of 1; blue circle represents adjusted OR from univariate regression analysis; grey area represents the 95% confidence interval

**Supplemental Figure 5. Multivariate ChemRICH enrichment plots depicting all pathways identified at A) 10-13 and B) 16-19 weeks of gestation^1,2^**

**
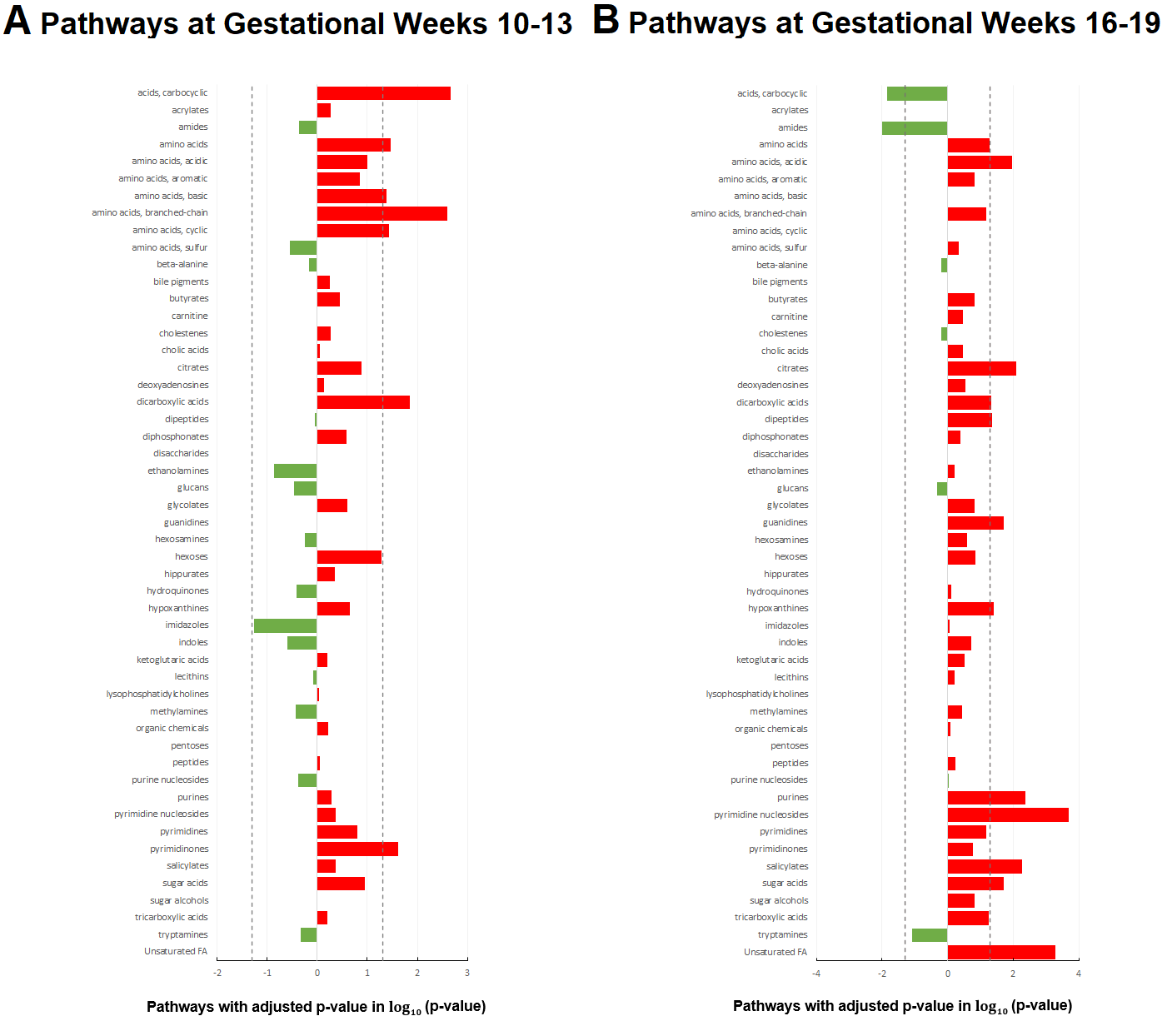

^1^**Red bars, upregulation; green bars, downregulation

**^2^**P <0.05 corresponds to −log_10_ of P-value >1.3 for upregulated pathways and log_10_ of P-value <-1.3 for downregulated pathways

| **Supplemental Table 3. The putative pathways linking microbiome-derived metabolites at 10-13 and 16-19 weeks of gestation (GW) to risk of gestational diabetes using the multivariate ChemRICH analysis** | | | | | | |
| --- | --- | --- | --- | --- | --- | --- |
| **Time Window** | **Pathway** | **P-value^1^** | **FDR** | **Hits** | **Metabolite Hits** | **Key Metabolite^2^** |
| **Gestational Weeks 10-13** | **Inverse Association**  None | **–** | **–** | **–** | **–** | **–** |
|  | **Positive Association** |  |  |  |  |  |
|  | Acids, carbocyclic | 0.0022 | **0.026** | 3 | 3-(4-hydroxyphenyl)propionic acid, 4-hydroxyphenylacetic acid, hydrocinnamic acid | 4-hydroxyphenyl  acetic acid |
|  | Amino acids | 0.034 | 0.130 | 7 | Citrulline, kynurenine, alanine, serine, glycine, threonine, beta-alanine | Citrulline |
|  | Amino acids, basic | 0.042 | 0.130 | 6 | Arginine, ornithine, l-glutamine, glutamine, asparagine, lysine | Arginine |
|  | Amino acids, branched-chain | 0.0025 | **0.026** | 3 | Leucine, isoleucine, valine | Isoleucine |
|  | Amino acids, cyclic | 0.037 | 0.130 | 4 | Histidine, oxoproline, trans-4-hydroxy-l-proline, proline | Histidine |
|  | Dicarboxylic acids | 0.014 | 0.095 | 8 | Citramalic acid, succinic acid, glutaric acid, 2,6-diaminopimelic acid, alpha-aminoadipic acid, pimelic acid, fumaric acid, malic acid | Citramalic acid |
|  | Pyrimidinones | 0.024 | 0.130 | 3 | 5-Methylcytosine, uracil, thymine | 5-Methylcytosine |
| **Gestational Weeks 16-19** | **Inverse Association**  Amides | 0.01 | 1 | 1 | Allantoic acid | Allantoic acid |
|  | **Positive Association** |  |  |  |  |  |
|  | Acids, carbocyclic | 0.014 | 0.098 | 3 | 3-(4-hydroxyphenyl)propionic acid, 4-hydroxyphenylacetic acid, hydrocinnamic acid | Hydrocinnamic acid |
|  | Amino acids, acidic | 0.011 | 0.098 | 4 | Glutamic acid, aspartic acid, l-glutamic acid, n-acetylaspartic acid | Glutamic acid |
|  | Citrates | 0.0083 | 1 | 1 | Citric acid | Citric acid |
|  | Dicarboxylic acids | 0.048 | 0.180 | 8 | Citramalic acid, alpha-aminoadipic acid, fumaric acid, succinic acid, pimelic acid, malic acid, 2,6-diaminopimelic acid, glutaric acid | Alpha-aminoadipic  acid |
|  | Dipeptides | 0.043 | 1 | 1 | Cysteine-glycine | Cysteine-glycine |
|  | Guanidines | 0.019 | 1 | 1 | Creatine | Creatine |
|  | Hypoxanthines | 0.039 | 1 | 1 | Hypoxanthine | Hypoxanthine |
|  | Purines | 0.0043 | 1 | 1 | Adenine | Adenine |
|  | Pyrimidine nucleosides | 0.0002 | 1 | 1 | Uridine | Uridine |
|  | Salicylates | 0.0053 | 1 | 1 | Salicylic acid | Salicylic acid |
|  | Sugar acids | 0.02 | 0.110 | 3 | Galactonic acid, glyceric acid, saccharic acid | Galactonic acid |
|  | Unsaturated fatty acids | 0.00053 | **0.011** | 8 | Homo-gamma-linolenic acid, oleic acid, eicosatrienoic acid, linoleic acid (positive), linoleic acid (negative), arachidonic acid, docosahexaenoic acid, linolenic acid | Oleic acid |
| **Changes from**  **Gestational Weeks 10-13 to 16-19** | **Inverse Association**  None | **–** | **–** | **–** | **–** | **–** |
|  | **Positive Association** |  |  |  |  |  |
|  | Amino acids, sulfur | 0.036 | 0.25 | 6 | Alpha-keto-gamma-(methylthio)butyric acid, DL-cystine, cysteine, methioninesulfoxide, homocysteine, methionine | Alpha-keto-gamma-(methylthio)butyric acid |
|  | Guanidines | 0.02 | 1 | 1 | Creatine | Creatine |
|  | Hexoses | 0.026 | 0.25 | 3 | Fructose, glucose, fucose | Fructose |
|  | Purines | 0.016 | 1 | 1 | Adenine | Adenine |
|  | Salicylates | 0.035 | 1 | 1 | Salicylic acid | Salicylic acid |
|  | Unsaturated fatty acids | 0.0005 | **0.011** | 8 | Homo-gamma-linolenic acid, oleic acid, eicosatrienoic acid, linoleic acid (positive), linoleic acid (negative), arachidonic acid, docosahexaenoic acid, linolenic acid | Eicosatrienoic acid |
| **^1^**The P value of each metabolite pathway was obtained by the Kolmogorov-Smirnov test after false discovery rate adjustment  **^2^**The most significant microbiome-derived metabolite within that pathway | | | | | | |

| **Supplemental Table 4: Predictive performance of multi-metabolite panels at 10-13 weeks and 16-19 weeks of gestation beyond conventional risk factors for gestational diabetes using LASSO regression models** | | | | | | |
| --- | --- | --- | --- | --- | --- | --- |
|  | **Discovery Set^1^** | | **Validation Set 1^2^** | | **Validation Set 2^3^** | |
|  | PPV**^*^** | NPV**^*^** | PPV | NPV | PPV | NPV |
| **Gestational Weeks 10-13** |  |  |  |  |  |  |
| Model 1**^4^** | 0.500 | 0.763 | 0.444 | 0.906 | 0.519 | 0.863 |
| Model 2**^5^** | 0.815 | 0.877 | 0.201 | 0.955 | 0.784 | 0.912 |
| Model 3**^6^** | 0.846 | 0.735 | 0.775 | 0.974 | 0.968 | 0.932 |
| **Gestational Weeks 16-19** |  |  |  |  |  |  |
| Model 1**^4^** | 0.529 | 0.770 | 0.467 | 0.926 | **–** | **–** |
| Model 2**^7^** | 0.596 | 0.851 | 0.233 | 0.937 | **–** | **–** |
| Model 3**^6^** | 0.568 | 0.851 | 0.500 | 0.931 | **–** | **–** |
| LASSO: least absolute shrinkage and selection operator; NPV: negative predictive value; PPV: positive predictive value | | | | | | |
| **^1^**Discovery set was a matched case-control study of 91 GDM cases and 180 non-GDM controls in the Pregnancy Environment and Lifestyle Study (PETALS) cohort | | | | | | |
| **^2^**Validation set 1 was a random sample of 42 GDM cases and 372 non-GDM controls from the PETALS cohort | | | | | | |
| **^3^**Validation set 2 included 35 GDM cases and 70 non-GDM controls from the GestationaL Weight Gain and Optimal Wellness (GLOW) randomized controlled trial; validation set 2 only applicable for gestational weeks 10-13 | | | | | | |
| **^4^**Model 1 included conventional risk factors: age at delivery, race/ethnicity, pre-pregnancy body mass index, nulliparity, pre-existing hypertension, family history of diabetes, gestational age and fasting status at the respective clinic visit, and fasting serum glucose values | | | | | | |
| **^5^**Model 2 included a 35-metabolite panel selected by LASSO regression at gestational weeks 10-13 (creatinine, homocysteine, citric acid, tryptophan, glycocholic acid, 4-hydroxyphenylacetic acid, citrulline, creatine, kynurenine, 5-methylcytosine, alanine, alpha-aminoadipic acid, 2,6-diaminopimelic acid, xanthine, eicosatrienoic acid, lactic acid, saccharic acid, 5,6-dihydrouracil, DL-cystine, trimethylamine-N-oxide, L-glutamine, urea, alpha-keto-gamma-(methylthio)butyric acid, 3-hydroxybutyrylcarnitine, fructose, 2-hydroxybutanoic acid, beta-alanine, thymine, 4-imidazoleacrylic acid, lysophosphatidylethanolamine 18:0, trans-4-hydroxy-l-proline, inosine, alpha-ketoglutarate, tryptamine, fucose) | | | | | | |
| **^6^**Model 3 included conventional risk factors in Model 1 and metabolites in Model 2 | | | | | | |
| **^7^**Model 2 included a 11-metabolite panel selected by LASSO regression at gestational weeks 16-19 (citric acid, uridine, arachidic acid, hydrocinnamic acid, tryptophan, lysophosphatidylethanolamine 18:0, glucose, homocysteine, 3-hydroxybutyrylcarnitine, lysophosphatidylethanolamine 16:0, linolenic acid) | | | | | | |

**Supplemental Figure 6: Model optimization of LASSO regression models for the selection of multi-metabolite panels at A) 10-13 and B) 16-19 weeks of gestation in the discovery set**

Dotted lines on the right highlighted A) 35 microbiome-derived metabolites**^1^** and B) 11 microbiome-derived metabolites**^2^** and indicated the marker for the area under the curve (AUC) within one standard error of the highest AUC; dotted lines on the left highlighted features that generated the highest AUC statistics

**
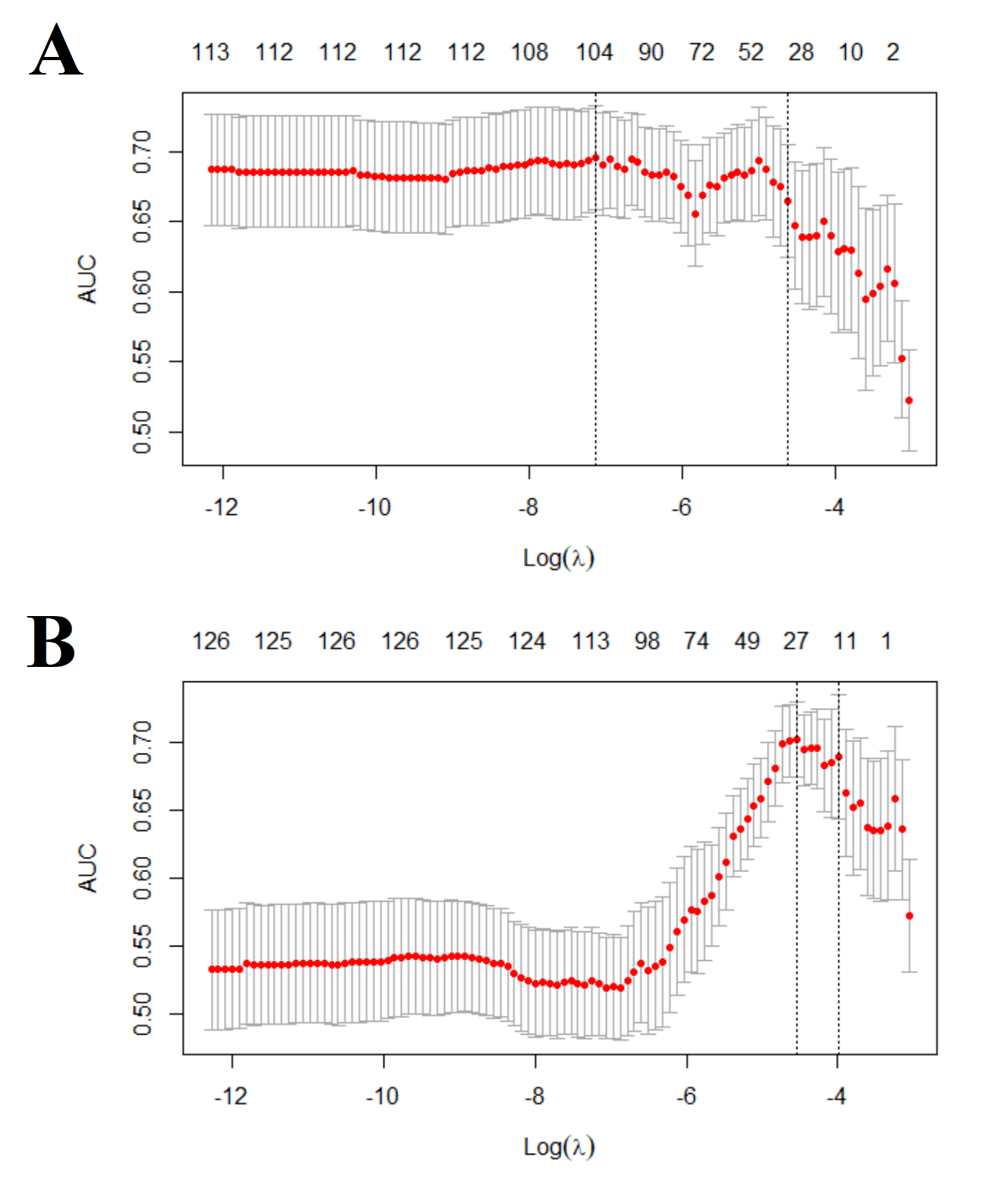
**

**^1^**The 35-metabolite panel included: creatinine, homocysteine, citric acid, tryptophan, glycocholic acid, 4-hydroxyphenylacetic acid, citrulline, creatine, kynurenine, 5-methylcytosine, alanine, alpha-aminoadipic acid, 2,6-diaminopimelic acid, xanthine, eicosatrienoic acid, lactic acid, saccharic acid, 5,6-dihydrouracil, DL-cystine, trimethylamine-N-oxide, L-glutamine, urea, alpha-keto-gamma-(methylthio)butyric acid, 3-hydroxybutyrylcarnitine, fructose, 2-hydroxybutanoic acid, beta-alanine, thymine, 4-imidazoleacrylic acid, lysophosphatidylethanolamine 18:0, trans-4-hydroxy-l-proline, inosine, alpha-ketoglutarate, tryptamine, fucose

**^2^**The 11-metabolite panel included: citric acid, uridine, arachidic acid, hydrocinnamic acid, tryptophan, lysophosphatidylethanolamine 18:0, glucose, homocysteine, 3-hydroxybutyrylcarnitine, lysophosphatidylethanolamine 16:0, linolenic acid

| **Supplemental Table 5: External validation of predictive multi-metabolite panels at 10-13 weeks and 16-19 weeks of gestation beyond conventional risk factors for gestational diabetes using LASSO regression models** | | |
| --- | --- | --- |
|  | **Validation Set 1^1^** | **Validation Set 2^2^** |
|  | **AUC (95% CI)** | |
| **Gestational Weeks 10-13** |  |  |
| Model 1**^3^** | 0.731 (0.638 – 0.824) | 0.717 (0.612 – 0.823) |
| Model 2**^4^** | 0.743 (0.662 – 0.824) | 0.936 (0.892 – 0.980) |
| Model 3**^5^** | 0.945 (0.900 – 0.990) | 0.987 (0.938 – 0.999) |
| P_Model 2 vs. 1_**^7^** | 0.7754 | <0.0001 |
| P_Model 3 vs. 2_**^7^** | <0.0001 | 0.0413 |
| P_Model 3 vs. 1_**^7^** | <0.0001 | <0.0001 |
| **Gestational Weeks 16-19** |  |  |
| Model 1**^3^** | 0.780 (0.688 – 0.871) | **–** |
| Model 2**^6^** | 0.681 (0.592 – 0.771) | **–** |
| Model 3**^5^** | 0.826 (0.748 – 0.905) | **–** |
| P_Model 2 vs. 1_**^7^** | 0.4899 | **–** |
| P_Model 3 vs. 2_**^7^** | 0.0585 | **–** |
| P_Model 3 vs. 1_**^7^** | 0.1089 | **–** |
| LASSO: least absolute shrinkage and selection operator  **^1^**Validation set 1 was a random sample of 42 GDM cases and 372 non-GDM controls from the PETALS cohort  **^2^**Validation set 2 included 35 GDM cases and 70 non-GDM controls from the GestationaL Weight Gain and Optimal Wellness (GLOW) randomized controlled trial; validation set 2 only applicable for gestational weeks 10-13  **^3^**Model 1 included conventional risk factors: age at delivery, race/ethnicity, pre-pregnancy body mass index, nulliparity, pre-existing hypertension, family history of diabetes, gestational age and fasting status at the respective clinic visit, and fasting serum glucose values  **^4^**Model 2 included a 35-metabolite panel selected by LASSO regression at gestational weeks 10-13 (creatinine, homocysteine, citric acid, tryptophan, glycocholic acid, 4-hydroxyphenylacetic acid, citrulline, creatine, kynurenine, 5-methylcytosine, alanine, alpha-aminoadipic acid, 2,6-diaminopimelic acid, xanthine, eicosatrienoic acid, lactic acid, saccharic acid, 5,6-dihydrouracil, DL-cystine, trimethylamine-N-oxide, L-glutamine, urea, alpha-keto-gamma-(methylthio)butyric acid, 3-hydroxybutyrylcarnitine, fructose, 2-hydroxybutanoic acid, beta-alanine, thymine, 4-imidazoleacrylic acid, lysophosphatidylethanolamine 18:0, trans-4-hydroxy-l-proline, inosine, alpha-ketoglutarate, tryptamine, fucose)  **^5^**Model 3 included conventional risk factors in Model 1 and metabolites in Model 2  **^6^**Model 2 included a 11-metabolite panel selected by LASSO regression at gestational weeks 16-19 (citric acid, uridine, arachidic acid, hydrocinnamic acid, tryptophan, lysophosphatidylethanolamine 18:0, glucose, homocysteine, 3-hydroxybutyrylcarnitine, lysophosphatidylethanolamine 16:0, linolenic acid)  **^7^**P-value was obtained by DeLong's test | | |
